# Rational and design of SATRACD study: detecting subclinical anthracycline therapy related cardiac dysfunction in low income country

**DOI:** 10.4314/ahs.v21i2.21

**Published:** 2021-06

**Authors:** Wanzhu Zhang, Feriel Azibani, Emmy Okello, James Kayima, Victoria Walusansa, Jackson Orem, Karen Sliwa

**Affiliations:** 1 Hatter Institute of Cardiovascular Research in Africa; 2 Uganda Heart Institute; 3 UMRS 942 Inserm, Paris 75010, France; 4 Makerere University, College of Health Science; 5 Uganda Cancer Institute

**Keywords:** SATRACD study, subclinical anthracycline therapy, cardiac dysfunction, low income country

## Abstract

**Background:**

Anthracycline therapy-related cardiac dysfunction (ATRCD) is the most notorious adverse side-effect of chemotherapy. It has become a significant cardiovascular health concern for long-term cancer survivors. With the emerging concept of subclinical ATRCD and newer diagnostictools (Speckle Tracking Echocardiography (STE) and biomarkers), detecting anthracycline cardiac toxicity at an early stage has become an important step to prevent severe cardiac dysfunction and improve the cardiovascular outcome in cancer survivors. Despite the increasing population at risk in sub-Saharan Africa (SSA), there is no contemporary data in Uganda to address the burden, pathogenesis and risk factors of subclinical ATRCD. This big gap in knowledge has led to a lack of local guidelines for monitoring and management of ATRCD.

**Methods:**

SATRACD (Detecting Subclinical Anthracycline Therapy Related Cardiac Dysfunction In Low Income Country) study is an observational prospective cohort study. Three hundred and fifty-three anthracycline naïve cancer patients will be recruited at baseline. Patients are followed up on completion of anthracycline-based chemotherapy and at 6 months after completion of anthracycline therapy. Data on demographics, cancer profile and clinical presentation will be collected at baseline. Comprehensive cardiac assessment will be performed at each visit, including electrocardiogram, conventional echocardiography, STE, cardiac and oxidative stress markers. We will be able to determine the incidence of subclinical and clinical ATRCD at 6 months after completion of anthracycline therapy, determine whether hypertension is a major risk factor for ATRCD, evaluate the role of conventional echocardiography parameters, and biomarkers for detecting subclinical ATRCD.

**Conclusion:**

This SATRACD study will provide contemporary data on Ugandan cancer patients who have subclinical and clinical ATRCD, help in the development of local strategies to prevent and manage ATRCD, and improve cardiovascular outcome for Ugandan cancer survivors.

## Introduction

Anthracyclines are potent antineoplastic agents with proven efficacy in the treatment of many pediatric-, adult-hematologic and solid organ cancers. Dose-dependent anthracycline therapy-related cardiac dysfunction (ATRCD) is the most common and well-studied chemotherapy-induced cardiovascular toxicity. It was first described in 1971 in 67 patients treated with Adriamycin for a variety of tumors.^1^ The clinical significance of anthracycline cardiotoxicity is increasing with larger numbers of cancer survivorship worldwide.^2^ The prevalence of late asymptomatic cardiac damage has been reported to be more than 57%, at a median of 6.4 years after treatment, among survivors of childhood cancers,^3^ and the incidence of symptomatic heart failure as high as 16%, 0.9 to 4.8 years after treatment.^4^ Risk of mortality from cardiac-related events is 8 times higher for long-term cancer survivors, who received anthracycline, than for the normal population.^5^, ^6^ Recent study findings suggest that anthracycline cardiotoxicity represents a continuum that begins with subclinical myocardial cell injury, followed by an early asymptomatic decline in left ventricular ejection fraction (LVEF) that can progress to symptomatic heart failure, if left untreated.^7^ Although, not all the subclinical left ventricular dysfunctions will become overt heart failure, however, these insults enhance cardiac susceptibility to further cardiovascular stresses (e.g. pregnancy, surgery, hypertension, etc) or injuries (radiation, ischemia, etc) and, ultimately, increase the risk of premature cardiovascular mortality.^8^ These findings have significant implications for the surveillance and management of anthracycline cardiotoxicity. A newer echocardiography (ECHO) technique, Global Longitudinal Strain (GLS) by STE, together with biomarkers (Troponin I), have gained importance in diagnosing subclinical ATRCD. These methods have been incorporated into current surveillance guidelines.^9^ Only a few studies reported the incidence of subclinical ATRCD. The Boyd, A. group reported subclinical ATRCD occurred in 22% of 140 breast cancer patients, who received anthracycline therapy.^10^ In another study of 159 patients receiving anthracycline, decreased GLS was found in 33% of the patients.^11^

Identification of patients at risk of subclinical or clinical ATRCD is also very important, except when implementing surveillance protocols. Pre-existing cardiac risk factors such as hypertension, diabetes mellitus, smoking, previous left ventricular dysfunction and coronary disease, have shown to increase the risk of anthracycline-related cardiac toxicity.^12^ Among these, hypertension, in particular, increases the risk of heart failure significantly in childhood cancer survivors who received anthracycline therapy.^13^ For adult cancer patients, hypertension and cancer often coexist in the same patient. In fact, high blood pressure is the most frequent co-morbid condition reported in cancer patients.^12^ The most common hypothesis of anthracycline cardiotoxic mechanisms is the formation of free radicals and superoxides, which lead to oxidative stress.^14^–^17^ Cardiac cells are more susceptible to free radical damage. Hypertension not only promotes cardiac remodeling, but also enhances oxidative stress.^18^ This implies that hypertension may serve as a significant risk factor for the development of anthracycline cardiotoxicity.

Cancer is emerging as a major public health problem in Sub-Saharan Africa (SSA), due to population ageing and growth.^19^ The growing prevalence of cancer in SSA determines the major role of anthracycline in cancer treatment in SSA.^20^ Black race was found to be a risk factor for developing ATRCD in both childhood cancer survivors^21^ and adult cancer patients.^22^ Furthermore, the higher prevalence of hypertension and hypertensive heart disease in Black patients^23^ may make hypertension a significant risk factor for developing ATRCD in the SSA population.

## Rationale

Despite the potential population at risk in SSA, there is a noted paucity of information on the burden of anthracycline-induced cardiotoxicity and related predictors among adult cancer patients receiving anthracycline chemotherapy. Lack of STE modality and expertise is a major obstacle for SSA countries to detect subclinical ATRCD. When STE is not available, conventional ECHO parameters, which measure the longitudinal motion of the left ventricle (e.g. mitral annular plane systolic exertion(MAPSE), peak systolic mitral annular velocity by tissue doppler (S'), may potentially be useful.^9^ However, their role in detecting subclinical ATRCD have not been studied. Despite the hypothesis of oxidative stress on the mechanism of anthracycline cardiotoxicity, the association between the oxidative stress markers and cardiotoxicity has not yet been well studied in African adult cancer patients, nor has the role of oxidative stress markers on the diagnosis of subclinical ATRCD.

In Uganda, heart failure caused by ATRCD is not uncommon. Patients usually present with advanced heart failure and carry a poor prognosis. In the Uganda Cancer Institute, 60% of the patients receive anthracycline therapy, but the majority of patients only receive screening ECHO prior to therapy. Follow-up cardiac evaluation is not routinely done. This may leave many anthracycline-treated patients with undetected asymptomatic subclinical cardiac dysfunction at risk of overt heart failure when they encounter another cardiovascular risk later in life.

Our knowledge of anthracycline cardiotoxicity derives mainly from data in the developed world.

To date, there are no contemporary data in Uganda to address the burden, pathogenesis and risk factors of subclinical ATRCD. This big gap in knowledge has led to a lack of local guidelines for monitoring and management of ATRCD.

Detecting ATRCD at the subclinical stage is very important for the long-term outcome of cancer survivors. Therefore, this study aims to fill the gaps in knowledge in Ugandan cancer patients who are treated with anthracycline, and to understand the disease better by studying the burden, risk factors and pathogenesis. The study will also identify other available conventional ECHO parameters and oxidative stress makers that are able to detect or rule out subclinical ATRCD. Therefore, the results of the study will promote the application of available resources in cardio-oncology clinical practice, help to establish national guidelines on cardiac monitoring and management of patients who receive anthracycline therapy.

## Methods

### Study design

This is a prospective study with two parallel Cohorts (Cohorts 1 and 2). Cohort 1 consecutively recruit 300 adult non-hypertensive cancer patients who receive anthracycline therapy. The time point prior to receiving anthracycline therapy (anthracycline naïve) is defined as baseline. The patient's demographic data, cancer diagnosis, dosage of anthracycline, past medical history, symptoms, physical examinations, ECG, ECHO and laboratory data are collected at baseline. Patient follow-ups are performed at the completion of chemotherapy, and 6 months after completion of chemotherapy. Data on symptoms, physical examinations, ECG, ECHO and blood test are collected at each visit.

Cohort 2 recruit 53 adult hypertensive cancer patients. The patient's demographic data, cancer diagnosis, dosage of anthracycline, past medical history, symptoms, physical examinations, ECG, ECHO and laboratory data are collected at baseline. Patient follow- ups are performed at the completion of the chemotherapy, and 6-months after completion of chemotherapy. Data of symptoms, physical examinations, ECG, ECHO and blood test are collected at each visit.

Objectives. Our objectives are as follow:

1. To determine the incidence of subclinical and clinical ATRCD in cancer patients who receive anthracycline therapy
2. To compare the changes of GLS value between hypertensive and non-hypertensive cancer patients at completion of anthracycline therapy
3. To determine the correlation of conventional ECHO parameters (e.g. MAPSE, S') with GLS, and their ability to diagnose or rule out subclinical ATRCD
4. To determine the correlation of oxidative stress with cardiac function (GLS value) in patients who receives anthracycline therapy and their role in diagnosing subclinical ATRCD.

We hypothesized that hypertension would be a significant risk factor for developing cardiotoxicity in Ugandan cancer patients treated with anthracycline. Conventional ECHO parameters and oxidative stress markers may play important roles in detecting subclinical ATRCD in Ugandan cancer patients.

### Study site and population

The patient screening site is at the outpatient clinic of the Uganda Cancer Institute (UCI). Patient recruitment and data collection is at the Uganda Heart Institute (UHI). UCI is a specialized national referral oncology center in Kampala, Uganda. The center receives about 1,000 new cancer patients referred for chemotherapy annually. Currently, it has a bed capacity of about 100 beds with 8 specialist medical oncologists, 2 pharmacists, 10 medical officers and 50 trained oncology nurses. About 60% of patients attending UCI receive anthracycline therapy. Adult patients who receive anthracycline, with expected survival longer than two years, are referred to UHI for re-screening of eligibility. Blood samples collected from patients will be stored and shipped to the Hatter Institute for Cardiovascular Research in Africa in Cape Town, where biomarker analysis will be carried out.

### Inclusion criteria and Exclusion criteria

#### Clinical intake protocol

The following data will be collected from all enrolled subjects:

- Patient demographics: age, gender, tribe, district
- Cancer profile: Cancer diagnosis and stage, type of anthracycline and accumulated dosage*, other medication*
- Past medical and social history: hypertension, diabetes mellitus, coronary heart disease, renal disease, HIV, smoking, alcohol intake, any other chronic illness
- Family history of heart disease or cancer
- Current clinical presentation*: symptoms of heart failure, NYHA class, heart failure stage
- Physical examination*: weight, height, temperature, blood pressure**, heart rate, SaO2, signs of heart failure (raised JVP, edema, rails, etc)

The following investigative tests will be performed:

- 12-lead resting Electrocardiogram*
- Comprehensive Echocardiography*: using 2D, M-mode, Doppler, tissue Doppler, Speckle Tracking Echocardiography (STE) image to assess cardiac structure, function and hemodynamics.
- Blood test*: CBC, LFTs, RFTs, FBS, Fasting lipid profile, Troponin I, N-pro BNP, myeloperoxidase (MOP).

*: Will be collected at each visit

**: Patient's left arm blood pressure will be taken at sitting position, using “Omron” digital blood pressure machine, after resting for 5 minutes. High blood pressure will be quantitatively defined as systolic blood pressure > 140mmHg or diastolic pressure > 90mmHg.

### Diagnosis of subclinical and clinical ATRCD

Subclinical ATRCD and clinical ATRCD are diagnosed using criteria recommended by the American Society of Echocardiography and the European Association of Cardiovascular Imaging:9

### Diagnostic criteria for subclinical ATRCD:

- LVEF≥53%

and

- A relative percentage decrease of GLS >15%, compared with baseline, and /or
- TnI become positive during follow up

Diagnostic criteria for clinical ATRCD:

- Decrease in LVEF of >10 percentage points, to a value <53%.

### Echocardiography protocol and equipment

Transthoracic echocardiograms images are acquired using the Vivid E9 (GE Healthcare), conducted by two formally trained cardiologists (including PI). All echocardiograms are analyzed by a single observer (PI). LVEF is calculated from the apical 4- and 2-chamber views using a modified Simpson biplane method. ATRCD is defined as a reduction in LVEF of >10%, to a value of <53%, as per guidelines.9 Speckle tracking strain analysis is used to measure global and regional longitudinal systolic strain from apical four-, two- and three-chamber views. Regional longitudinal strain is demonstrated on a Bull's eye diagram. MAPSE is measured by m-mode obtained from apical four camber view. Average of septal and lateral MAPSE is calculated. Tissue Doppler-derived indices is measured using the apical four-chamber view. Peak systolic mitral annular velocities (S') is calculated by averaging septal and lateral mitral annular velocities.

### Biomarker measurements

In all patients, blood samples are obtained from venal puncture. Cardiac troponin I is measured as part of the clinical routine. The blood sample is collected in an EDTA tube and SST tube and immediately centrifuged. Plasma and serum are stored at -80 degree Celsius until assayed. The stored blood samples will be transported on dry ice and shipped to the Hatter Institute for Cardiovascular Research in Africa, where the assay for NT-pro BNP and markers for oxidative stress markers will be carried out. The shipment of the samples and biomarker analyses will only be done on samples of patients who have a complete data set, including all the time points (baseline + two follow up visits.)

NT-proBNP plasma levels will be measured by electrochemiluminescence immunoassay (BNP Fragment EIA, Biomedica, Vienna, Austria). Oxidative stress markers, e.g. serum myeloperoxidase (MPO) will be investigated in our cohort. Serum MPO will be measured using the human MPO Instant ELISA (ThermoFisher Scientific, USA).

Appropriate dilution of the plasma or serum samples will be utilized for the assays, as required by the manufacturer of each ELISA kit, and each plasma or serum sample will be assayed in duplicate. The average of the two measurements will be utilized to calculate the levels of each of the biomarkers from the standard curve established with known positive standards supplied with each kit.

### Study Information Management

Study data is entered via specialized online study forms into a central database in REDCap. REDCap is a widely used research information system that facilitates formbased entry of study data with quality assurance tools to check the validity and consistency of results.

### Data analysis Plan

All continuous variables are expressed as a mean ± SD and categorical variables as a percentage.

Student's t-test, paired t-test, and Mann-Whitney U test will be used to compare continuous variables, where appropriate. Correlations will be assessed by Pearson's t-test. Significant relationships will then be examined by multivariate linear regression analysis. Receiver operating characteristic (ROC) curves will be plotted to examine the abilty of MAPSE and S' to detect subclinical ATRCD (objective 3). Data will be analyzed using SPSS version 17 and considered significant if p <0.05.

### Ethical consideration

Ethical approvals were obtained from School of Medicine Ethics and Research Committee, College of Health Sciences Makerere University (REC REF 2018-081), Uganda National Council of Science and Technology (HS220ES) and Faculty of Health Sciences Human Research Ethics Committee, University of Cape Town (HREC 054/2020sa)

All participants are provided with a written copy of the consent form. Any and all questions that participants have regarding the research will be addressed prior to signing of the consent form. Written informed consent in English or Luganda is collected from each participant.

### Status of the study

Between November 2018 and February 2020, 353 patients (age:43±12 years, 79% female) were enrolled at the baseline. There were 167 patients who were able to complete anthracycline-based chemotherapy and who had returned for the second follow-up visit by April 2020. Of these 167 patients, 36 were able to return for the third (last) follow-up visit by April 2020.

We expect to complete patient follow-up by the end of 2020 or early in 2021.

## Discussion

Anthracycline therapy-related cardiac dysfunction is a well-known adverse effect resulting from this potent anti-cancer regimen. Recently, the emerging concept of subclinical ATRCD and novel diagnostic tools made close cardiac surveillance highly important. To detect and treat ATRCD at the subclinical stage and improve the cardiovascular outcome for long-term cancer survivors have become one of the major goals of cardio-oncology practices worldwide. With the increased cancer burden and higher exposure to anthracycline in the SSA population, subclinical ATRCD should no longer being ignored. The SATRACD study is the first observational cohort study, using international guidelines, to define the burden and risk factors of subclinical ATRCD in a SSA country (Uganda). It evaluates the role of conventional ECHO parameters and biomarkers in detecting subclinical ATRCD. In this study, for the first time, STE is performed in Ugandan cancer patients. This will not only enable us to monitor patients more effectively, but will also initiate cardio-oncology practices in Uganda, promote capacity-building in cardiovascular medicine and initiate more research with STE at stage A and B heart failure and other heart diseases. Data from the SATRACD study will bridge the gap in knowledge of anthracycline cardiotoxicity in Uganda, and, furthermore, help to develop local guidelines on cardiac monitoring for early diagnosis and treatment of ATRCD. Following the findings of the SATRACD study, a further clinical trial may be designed to study the effectiveness of carvedilol on treating subclinical ATRCD in the future.

## Figures and Tables

**Figure I:**
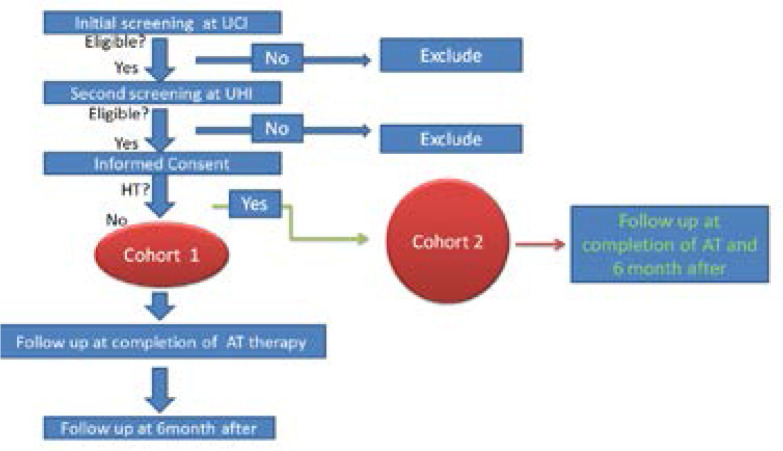
Diagram of the protocol

**Table I:** Inclusion and exclusion criteria

Cohort	Inclusion criteria	Exclusion criteria
1. 300 patients	Age ≥ 18 years Newly diagnosed cancer patients, before receiving anthracycline therapy	Baseline LVEF< 53% Patients with poor quality 2DE image. Advanced cancer patients with expectation survival less than two years Hypertension Patients who are unable to return for follow up-visits
2. 53 patients	Age ≥ 18 years Newly diagnosed cancer patients before receiving anthracycline therapy, who have high blood pressure or are taking antihypertensive medication.	Baseline LVEF < 53% Patients with poor quality 2DE image. Advanced cancer patients with expectation survival less than two year Patients who are unable to return for follow-up visits

**Table II:** Data collection and patients follow-up

Data	Baseline	Completion of anthracycline therapy	6 months after completion of anthracycline therapy
Demographics	X		
Cancer data	X	X	
Medical & social history	X		
Symptoms & physical examinations	X	X	X
ECG	X	X	X
ECHO	X	X	X
Lab test	X	X	X
